# Technical and imaging factors influencing performance of deep learning systems for diabetic retinopathy

**DOI:** 10.1038/s41746-020-0247-1

**Published:** 2020-03-23

**Authors:** Michelle Y. T. Yip, Gilbert Lim, Zhan Wei Lim, Quang D. Nguyen, Crystal C. Y. Chong, Marco Yu, Valentina Bellemo, Yuchen Xie, Xin Qi Lee, Haslina Hamzah, Jinyi Ho, Tien-En Tan, Charumathi Sabanayagam, Andrzej Grzybowski, Gavin S. W. Tan, Wynne Hsu, Mong Li Lee, Tien Yin Wong, Daniel S. W. Ting

**Affiliations:** 1 Singapore Eye Research Institute, Singapore National Eye Center, Singapore, Singapore; 2 Duke-NUS Medical School, Singapore, Singapore; 3 School of Computing, National University of Singapore, Singapore, Singapore; 4 Department of Ophthalmology, University of Warmia and Mazury, Olsztyn, Poland; 5 Institute for Research in Ophthalmology, Foundation for Ophthalmology Development, Poznan, Poland; 6 State Key Laboratory of Ophthalmology, Zhongshan Ophthalmic Center, Sun Yat-sen University, Guangzhou, China

**Keywords:** Population screening, Translational research

## Abstract

Deep learning (DL) has been shown to be effective in developing diabetic retinopathy (DR) algorithms, possibly tackling financial and manpower challenges hindering implementation of DR screening. However, our systematic review of the literature reveals few studies studied the impact of different factors on these DL algorithms, that are important for clinical deployment in real-world settings. Using 455,491 retinal images, we evaluated two technical and three image-related factors in detection of referable DR. For technical factors, the performances of four DL models (VGGNet, ResNet, DenseNet, Ensemble) and two computational frameworks (Caffe, TensorFlow) were evaluated while for image-related factors, we evaluated image compression levels (reducing image size, 350, 300, 250, 200, 150 KB), number of fields (7-field, 2-field, 1-field) and media clarity (pseudophakic vs phakic). In detection of referable DR, four DL models showed comparable diagnostic performance (AUC 0.936-0.944). To develop the VGGNet model, two computational frameworks had similar AUC (0.936). The DL performance dropped when image size decreased below 250 KB (AUC 0.936, 0.900, *p* < 0.001). The DL performance performed better when there were increased number of fields (dataset 1: 2-field vs 1-field—AUC 0.936 vs 0.908, *p* < 0.001; dataset 2: 7-field vs 2-field vs 1-field, AUC 0.949 vs 0.911 vs 0.895). DL performed better in the pseudophakic than phakic eyes (AUC 0.918 vs 0.833, *p* < 0.001). Various image-related factors play more significant roles than technical factors in determining the diagnostic performance, suggesting the importance of having robust training and testing datasets for DL training and deployment in the real-world settings.

## Introduction

Diabetic retinopathy (DR), is a major cause of blindness^1,2^. Cost-effective strategies for DR management includes routine screening using retinal photographs and having referable cases (typically moderate or worse DR and/or diabetic macular edema) managed by eye care specialists^3–5^. Recently, deep learning (DL) using convolutional neural networks (CNNs) has sparked tremendous interest in medicine^6^. In ophthalmology, many DL algorithms and systems have been reported to achieve robust performances in detecting various ocular diseases from retinal photographs^7–9^, especially for DR^10–13^. Despite substantial promise of DL technology, it is unclear what factors may influence the performance of a DL algorithm^14^. Currently, many research groups have developed different DL algorithms using different datasets and different techniques, and comprehensive guidelines on best practices are not yet available^8,9,15–20^.

There have been many studies, primarily in computer vision, exploring various factors that seek to optimize DL algorithms, albeit individually analyzed, thus making consistency difficult to achieve^21–25^. In addition, some factors involved in algorithm design such as input size and field have been overlooked and underestimated^26^. Some groups have looked at a compilation of technical factors postulated to be critical in the development of a DL algorithm in the clinical setting in detection of pathologies from radiological imaging^27–29^. However, these factors are largely domain specific (i.e., radiology)^27,30^. Thus, factors relevant in ophthalmology and particularly in the area of fundus imaging have yet to be explored.

The objective of this study is to systematically review current literature investigating possible factors that may influence the performance of a DL algorithm in detecting DR from fundus photographs. We then specifically addressed some of these factors that may impact on the performance of a DL algorithm. This study provides insights into technical and image-related factors that may impact future developments of DL systems for retinal image analysis, especially in context of tele-ophthalmology settings.

## Results

### Systematic review of literature

**Fig. 1:**
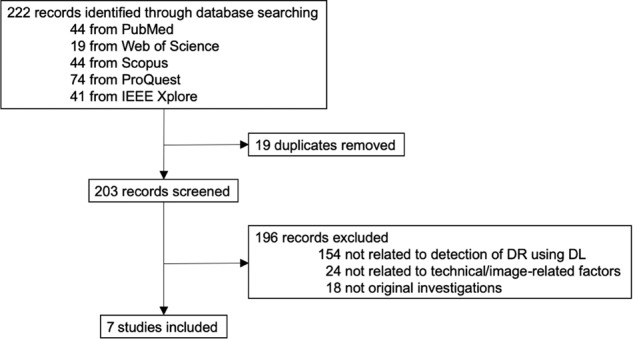
Study selection. Flowchart detailing the systematic literature review conducted to identify suitable studies that have evaluated technical and/or image-related factors that may influence the performance of a DL algorithm in detection of DR.

The results of our systematic review of literature are detailed in Fig. 1. Our search yielded 222 results, of which seven studies were identified to demonstrate evaluation of technical or image-related factors in DR detection by a DL algorithm. Table 1 displays the various factors and research questions addressed by the respective studies, demonstrating the focus on image-related factors by previous analyses, ranging from training dataset sizes to retinal camera specifications. Table 2 details the essential components, outcome measurements and implications of the articles included in the systematic review^9,15,25,31–34^.

**Table 1:** Technical and image-related challenges to development of deep learning algorithms for ocular disease detection.

Challenges		Research question	Paper addressing this question	Answer to research question
Technical	Newer convolutional neural networks with increasing number and complexity of layers may allow for greater depth of analysis but may intensify burden on hardware processing power and memory.	Does altering the convolutional neural network architecture affect performance?	Current paper	No. Different neural networks do not affect performance.
	Differences between computational frameworks based on flexibility, applicability, speed, ease of use, may affect choice.	Does altering the computational framework affect performance?	Current paper	No. Different computational frameworks do not affect performance.
Image-related	Lack of access to high quality retinal images due to poor fundus camera specifications, reduced storage space, or compression for tele-ophthalmology.	Does altering the level of compression of the input data affect performance?	Current paper	Yes. Reducing image size below 250 KB drops performance significantly.
	Different groups in various countries may possess datasets with varying number of field of fundus views due to disparities in protocols, resources, and manpower.	Does altering the number of fundus field of views of the input data affect performance?	Current paper	Yes. Performance drops in descending order from 7-field to 2-field to 1-field.
	The presence of cataract may impinge on proper visualization of the fundus and inaccurate diagnosis due to media opacity, light scatter and aberrancies.	Does previous cataract surgery affect performance?	Current paper	Yes. Presence of media opacity in phakic eyes reduces performance.
	The range of retinal cameras available to capture fundus images in terms of camera specifications, requirement for mydriasis, may provide variability in degree of field of view and image quality output.	Does altering the retinal cameras used affect performance?	Ting et al.^15^	No. Different retinal cameras do not affect performance.
	Ethnic differences in eyes exist that affect optical systems’ ability to capture the posterior pole and the identification of the norm (e.g. pigmentation, optic disc size, vasculature).	Do images from various ethnic groups affect performance?	Ting et al.^15^, Bellemo et al.^32^	No. Images from different ethnic groups do not affect performance.
	Different populations vary in prevalence rates of ocular disease, thus affecting the dataset used for validation and the utility of a clinical test deployed in that population.	Does deployment in populations with different disease prevalence rates affect performance?	Ting et al.^15^, Ting et al^34^.	No. Deployment in populations with different prevalence rates does not affect performance.
	Ocular diseases do not develop distinctly as many share similar risk factors and occur concurrently in the same patient, thus distinction between manifestations of different diseases is paramount.	Does concurrent related ocular diseases affect performance in detection of an individual disease?	Ting et al.^15^	No. Other existing diseases do not affect the algorithm’s ability to detect individual diseases accurately.
	The type of study (population-based, clinic-based or screening cohort) used to collect retinal images may influence the patient demographics of the datasets.	Does the type of studies affect the performance?	Ting et al.^15^	No. The type of study does not affect the performance.
	Different countries may use different reference standards for grading of diabetic retinopathy (e.g., grader or ophthalmologist), a product of resource allocation, expertise and training available.	Does the difference in reference standard used for labeling of images affect performance?	Ting et al.^15^	No. Different reference standards used do not affect the performance.
	Availability of large datasets in the target population may be scarce and insufficient for the training required for a highly performing algorithm.	Does a smaller dataset used for training affect the performance?	Gulshan et al.^9^, Burlina et al.^33^	Yes. Datasets that drop below 60,000 images produce large drops in performance.
	With large amount of images required for training, time constraints and reduced access to high quality retinal cameras may limit the use of large high resolution images for training of deep learning systems.	Does image size of the training dataset affect the performance?	Sahlsten et al.^25^	Yes. Increased resolution of training images produce better performance but increases training time.
	Mydriasis may provide greater visualization for photographic capture of the posterior pole, potentially influencing quality of fundus photographs.	Does mydriatic photographs improve performance compared to non-mydriatic images?	Gulshan et al.^9^, Bawankar et al.^31^	No. Mydriasis does not significantly improve performance.

**Table 2:** Characteristics of included studies in systematic review.

First author, reference	Factor addressed of training /testing dataset	Data points	Training dataset	Number of Images (training dataset)	Testing dataset	Number of images (testing dataset)	Outcome measures	Results						Implications
Gulshan^9^	Dataset size (% of total training dataset of 103,698) (Training)	0.2%	EyePACS	207	EyePACS	24,360	SP (at pre-set 97% SN)	SP 38%						60,000 Images may be the minimum training dataset size needed for maximum performance
		2%		2073				61%						
		10%		10,369				77%						
		20%		20,739				86%						
		30%		31,109				91%						
		40%		41,479				98%						
		50%		51,849				100%						
		60%		62,218				96%						
		70%		72,588				97%						
		80%		82,958				100%						
		90%		93,328				99%						
		100%		103,698				100%						
	Mydriasis (testing)	Mydriatic	EyePACS	128,175	EyePACS-1	4236	SN SP	SN 89.6%		SP 97.9%				Mydriasis may not be required for optimal performance
		Non-Mydriatic				4534		90.9%		98.5%				
		Both				8770		90.1%		98.2%				
Ting^15^	Retinal cameras (testing)	Canon	SiDRP	76,370	BES	1052	AUC SN SP	AUC 0.929	SN 94.4%			SP 88.5%		Different types of retinal cameras do not affect the performance
		Topcon			CUHK	1254		0.948	99.3%			83.1%		
		Carl Zeiss			HKU	7706		0.964	100%			81.3%		
		Fundus Vue			Guangdong	15,798		0.949	98.7%			81.6%		
	Study type (testing)	Clinic-based	SiDRP	76,370	CUHK	1254	AUC SN SP	AUC 0.948	SN 99.3%				SP 83.1%	The study type does not affect the performance in detection of disease
		Community-based			BES	1052		0.929	94.4%				88.5%	
		Population-based			Guangdong	15,798		0.949	98.7%				81.6%	
	Reference Standard (testing)	Retinal Specialists	SiDRP	76,370	CUHK	1254	AUC SN SP	AUC 0.948	SN 99.3%				SP 83.1%	If minimally professional graders with ≥7 years’ experience grade, performance may not be affected
		Ophthalmologists			BES	1052		0.929	94.4%				88.5%	
		Optometrists			HKU	7706		0.964	100%				81.3%	
		Graders			RVEEH	2302		0.983	98.9%				92.2%	
	Prevalence rate (testing)	5.5% (BES)	SiDRP	76,370	BES	1052	AUC SN SP	AUC 0.929	SN 94.4%				SP 88.5%	Lower prevalence rate does not greatly affect performance
		8.1% (SCES)			SCES	1936		0.919	100%				76.3%	
		12.9% (AFEDS)			AFEDS	1968		0.980	98.8%				86.5%	
	Concurrent diseases (testing)	Mixed pathologies	SiDRP	76,370	DR	37,001	AUC SN SP	AUC 0.936	SN 90.5%				SP 91.6%	Concurrent ocular pathologies in the same image does not affect the model’s detection of either disease
					AMD	773		0.942	96.4%				87.2%	
					Glaucoma	56		0.931	93.2%				88.7%	
	Ethnicity (testing)	Malay	SiDRP	76,370	SIMES	3052	AUC SN SP	AUC 0.889	SN 97.1%				SP 82.0%	Despite difference in the retina between ethnicities, this does not influence the performance in detection
		Indian			SINDI	4512		0.917	99.3%				73.3%	
		Chinese			SCES	1936		0.919	100%				76.3%	
		African American			AFEDS	1968		0.980	98.8%				86.5%	
		White			RVEEH	2302		0.983	98.9%				92.2%	
		Hispanic			Mexico	1172		0.950	91.8%				84.8%	
Bawankar^31^	Mydriasis (testing)	Non-mydriasis (vs ETDRS mydriatic reference standard)	Eye-PACS1, India	80,000	India	1084	SN SP	SN 91.2%		SP 96.9%				Despite no mydriasis of testing dataset, the DLS was able to perform highly when compared to mydriatic 7-field ETDRS grading reference standard
Burlina^33^	Dataset size (training)	Real	AREDS	119,090	AREDS	13,302	AUC AC	AUC 0.971			AC 91.1%			Creating proxy datasets using GANs may provide a solution to those with limited access to large number of images
		Synthetic	Image generated with GANs	119,090				0.924			82.9%			
Sahlsten^25^	Image pixel size (training)	256×256	Digifundus Ltd (Finland)	24,806	Digifundus Ltd (Finland)	7118	AUC	AUC0.961						Training with higher resolution images may improve performance
		299×299		24,806				0.970						
		512×512		24,806				0.979						
		1024×1024		24,806				0.984						
		2095×2095		24,806				0.987						
Bellemo^32^	Ethnicity (testing)	African	SiDRP	76,370	Zambia	4504	AUC SN SP	AUC 0.973	SN 92.3%				SP 89.0%	Differences in ethnicity between training and testing dataset does not affect performance
Ting^34^	Prevalence rate (testing)	4.1% (VTDR)	SiDRP	76,370	Pooled dataset (SiDRP, SIMES, SINDI, SCES, BES, AFEDS, CUHK, DMP)	93,293	AUC	AUC 0.950						Prevalence rate of diseases may be estimated accurately by DLS
		6.5% (RDR)						0.963						
		15.9% (ADR)						0.863						

*AUC* area under curve of receiver operating curve, *AC* accuracy, *SN* sensitivity, *SP* specificity, *EyePACS* Eye Picture Archive Communication System, *SiDRP* Singapore’s National Integrated Diabetic Retinopathy Screening Program, *BES* Beijing Eye Study, *CUHK* Chinese University Hong Kong, *HKU* Hong Kong University, *RVEEH* Royal Victoria Eye and Ear Hospital, *AFEDS* African American Eye Disease Study, *SCES* Singapore Chinese Eye Study, *SIMES* Singapore Malay Eye Study, *SINDI* Singapore Indian Eye Study, *DMP* Diabetes Management Project Melbourne, *DLS* Deep Learning System, *ETDRS* Early Treatment Diabetic Retinopathy Study, *AREDS* Age-Related Eye Disease Study, *DR* diabetic retinopathy, *AMD* age-related macular degeneration, *VTDR* vision threatening diabetic retinopathy, *RDR* reference diabetic retinopathy, *ADR* any diabetic retinopathy, *GAN* Generative Adversarial Network.

### Patient demographics and disease breakdown of datasets

Out of a total of 38,185 eyes included in the Singapore’s national integrated Diabetic Retinopathy Screening Program (SiDRP) primary testing dataset, 8.4% had referable DR (*n* = 3192). This proportion is similarly reflected in the SiDRP source testing dataset used, with 3.8% referable DR (*n* = 1373) in 35,948 eyes. Comparably, this is likewise seen in the external testing datasets, with African American Eye Disease Study (AFEDS) having 6.4% referable DR (*n* = 90) within a total of 1403 eyes and the Singapore Epidemiology of Eye Diseases (SEED) dataset having 8.5% referable DR (*n* = 415) within a total of 4910 eyes. These datasets, including detailed demographic characteristics, and breakdown into training and testing subsets have been previously published^15^.

### Technical factors: effect on performance

**Fig. 2:**
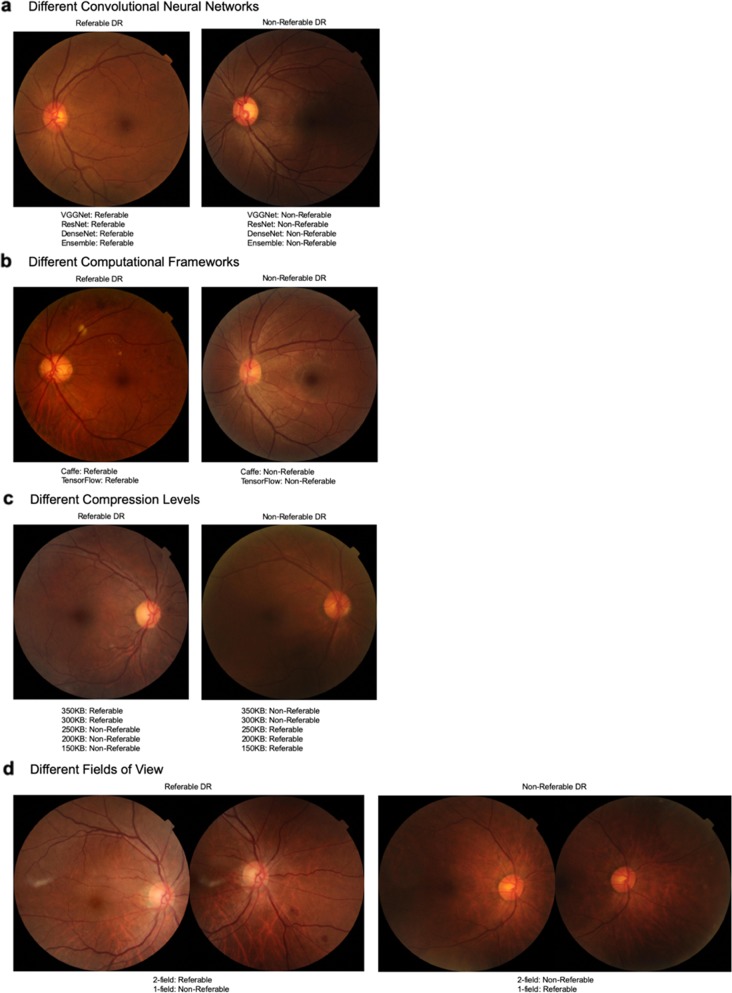
Retinal image examples. **a** Our results showed that using different CNNs show complementary classification of referable or non-referable DR, and these two images exhibit this agreement. **b** Using either computational framework similarly does not affect performance significantly as many images such as those depicted above are correctly classified as non-referable or referable DR by either framework. **c** Altering the image compression level does affect the DL model’s performance significantly beyond the threshold of 250 KB with a drop in sensitivity and specificity. These two photographs illustrate examples where a referable DR image is correctly identified as referable by the DL model when mild compression is introduced (i.e., a true positive case), but with further compression beyond 250 KB, this is misclassified as non-referable (i.e., a false negative case). This supports the drop in sensitivity beyond the 250 KB threshold. Similarly, this is demonstrated for a case of non-referable DR, where higher compression of the image causes a previously correctly classified image to subsequently be incorrect (i.e., a previously true negative result, now falsely classified as positive with disease), supporting the drop in specificity. **d** Another amendment to the image characteristics, in this case the field of view, showed reduced sensitivity and specificity when using 1-field instead of 2-field images. This example of referable DR had significant lesions present in the inferior-nasal quadrant, which were likely to be missed if using simply a macula-centered image, supporting the drop in sensitivity with the solitary use of 1-field images. Conversely, this example of healthy retina captured some dust particles in the superior and inferior nasal quadrant that might have inadvertently been misinterpreted by the DL algorithm as a lesion, prompting the misclassification as referable DR, thus supporting the drop in specificity.

Diagnostic performances of the DL algorithms using different CNNs and computational frameworks are shown in Table 3. Regardless of the CNN or computational framework employed in this study, all the DL algorithms were able to achieve high diagnostic performance—area under the receiver operating curve (AUC) ranged from 0.936 to 0.944, and sensitivities and specificities all exceeded 90% at the chosen classification thresholds. Newer CNNs showed no significant improvement in diagnostic performance. Compared to the oldest CNN VGGNet (AUC 0.938), ResNet (AUC 0.936; *P* = 0.581), and DenseNet (AUC 0.941; *P* = 0.410) have similar performance to VGGNet despite their increasing complexity in layers. However, an Ensemble of the three networks showed higher performance at detecting referable DR (AUC 0.944; *P* = 0.02). Sensitivities ranged from 91.9 to 94.0% with Ensemble producing the highest sensitivity, and specificities narrowly ranged from 90.7 to 91.0%. To illustrate this consistency in the performance between the different CNNs, an example is shown in Fig. 2a.

**Table 3:** Effect of technical factors specifically convolutional neural networks and computational framework.

			Convolutional neural networks				Computational frameworks	
			VGGNet	ResNet	DenseNet	Ensemble	Caffe	TensorFlow
SiDRP	Value (95% CI)	AUC	0.938 (0.929–0.945)	0.936 (0.927–0.944)	0.941 (0.933–0.947)	0.944 (0.938–0.950)	0.936 (0.927–0.944)	0.938 (0.929–0.945)
		*P* value for AUC comparison	Reference	0.581	0.410	0.02	Reference	0.736
		Sensitivity	92.1% (89.2–94.5%)	91.9% (88.9–94.3%)	92.8% (90.0–95.1%)	94.0% (91.3–96.0%)	90.5% (87.3–93.1%)	92.1% (89.2–94.5%)
		Specificity	91.0% (90.7–91.3%)	90.9% (90.6–91.2%)	90.9% (90.6–91.2%)	90.7% (90.4–91.0%)	91.9% (91.6–92.2%)	91.0% (90.7–91.3%)

*P* value was calculated by bootstrap method.

Dataset used for evaluation of different computational frameworks and convolutional neural networks is Singapore integrated Diabetic Retinopathy Programme (SiDRP) 2014 to 2015. During the evaluation of the impact of the convolutional neural network (CNN) on the DL algorithm performance, the computational framework was controlled for by using TensorFlow for fair comparison. Similarly, during the evaluation of different computational frameworks, the convolutional neural network controlled was controlled for by using VGGNet for isolation of independent variables.

*AUC* area under receiver operating curve, *CI* confidence Interval, *SiDRP* Singapore integrated Diabetic Retinopathy Programme.

Similarly, changing the computational frameworks used did not result in significant differences in diagnostic performance. Caffe and TensorFlow showed comparable performances with similar AUCs (0.936 vs 0.938; *P* = 0.736), sensitivities (90.5% vs 92.1%) and specificities (91.9% vs 91.0%). An example is displayed in Fig. 2b.

### Image-related factors: effect on performance

Diagnostic performances of the DL algorithms using different image sizes, numbers of fields, and prior cataract surgery are shown in Tables 4–6, respectively. Variation of these image characteristics had significant effects on diagnostic performance of the DL algorithms. With progressive reduction in image size from the original 350kilobytes (KB) to 300, 250, 200, and 150 KB, AUC dropped progressively from 0.936 to 0.921, 0.900, 0.896, and 0.891 respectively with decreases amounting to statistical significance below 250 KB in size (*P* < 0.001) and falling below the AUC 0.9 mark. Although sensitivities were maintained high, ranging from 83.5 to 90.5%, due to the previously fixed operating point, specificities dropped culminating in a specificity of 72.4% when images of 150 KB in size were used. Figure 2c illustrates this threshold with examples of retinal images of referable DR that were identified correctly as referable in minimal compression, but subsequently misclassified as non-referable when compression increased beyond 250 KB in image size, and vice versa.

**Table 4:** Effect of image-related factors specifically compression levels.

			Compression level – image file size				
			350 KB	300 KB	250 KB	200 KB	150 KB
SiDRP	Value (95% CI)	AUC	0.936 (0.927–0.944)	0.921 (0.908–0.932)	0.900 (0.885–0.913)	0.896 (0.881–0.910)	0.891 (0.876–0.905)
		*P* value for AUC comparison	Reference	0.261	<0.001	<0.001	<0.001
		Sensitivity	90.5% (87.3–93.1%)	85.9% (82.2–89.0%)	83.5% (79.7–86.9%)	85.6% (81.9–88.8%)	90.5% (87.3–93.1%)
		Specificity	91.9% (91.6–92.2%)	92.5% (92.3–92.8%)	88.8% (88.5–89.2%)	85.3% (84.9–85.7%)	72.4% (71.9–72.8%)

*P* value was calculated by bootstrap method, taking 350 KB as the reference for comparison against.

Dataset used for evaluation of different compression levels is Singapore integrated Diabetic Retinopathy Programme (SiDRP) 2014 to 2015.

*AUC* area under receiver operating curve, *CI* confidence interval, *KB* kilobytes, *SiDRP* Singapore integrated Diabetic Retinopathy Programme.

**Table 5:** Effect of image-related factors specifically fundus fields of view.

			Fundus fields of view		
			7-field (ETDRS standard)	2-field (Optic disc and macula-centered)	1-field (Macula-centered)
SiDRP	Value (95% CI)	AUC		0.936 (0.927–0.944)	0.908 (0.894–0.920)
		*P* value for AUC comparison		Reference	<0.001
		Sensitivity		90.5% (87.3–93.1%)	89.4% (86.0–92.2%)
		Specificity		91.9% (91.6–92.2%)	89.4% (89.0%–89.7%)
AFEDS	Value (95% CI)	AUC	0.949 (0.923–0.968)	0.911 (0.877–0.937)	0.895 (0.852–0.931)
		P value for AUC comparison	Reference	<0.001	<0.001
		Sensitivity	90.0% (81.9–95.3%)	82.6% (72.9–89.9%)	78.4% (67.3–87.1%)
		Specificity	86.5% (84.6–88.3%)	84.4% (82.3–86.3%)	86.1% (84.0–88.0%)

*P* value was calculated by bootstrap method.

Datasets used for evaluation of different fundus field of views were Singapore integrated Diabetic Retinopathy Programme (SiDRP) 2014 to 2015 to evaluate 2-field and 1-field, and African American Eye Disease Study to evaluate 7-field ETDRS standard retinal images in addition to 2-field and 1-field.

*AUC* area under receiver operating curve, *CI* confidence interval, *ETDRS* Early Treatment Diabetic Retinopathy Study, *SiDRP* Singapore’s national integrated Diabetic Retinopathy Screening Program, *AFEDS* African American Eye Disease Study.

**Table 6:** Effect of image-related factors specifically previous cataract surgery.

			Lens Status	
			Phakic	Pseudophakic
SEED	Value (95% CI)	AUC	0.833 (0.811–0.853)	0.918 (0.887–0.940)
		*P* value for AUC comparison	Reference	<0.001
		Sensitivity	91.1% (84.6–95.5%)	93.4% (85.3–97.8%)
		Specificity	76.1% (73.8–78.3%)	84.2% (81.4–86.8%)

*P* value was calculated by bootstrap method, using the phakic eyes as the standard.

Dataset used for evaluation of phakia compared to pseudophakia is Singapore Epidemiology of Eye Diseases study, which comprises of Singapore Malay Eye Study, Singapore Indian Eye Study and Singapore Chinese Eye Study.

*AUC* area under receiver operating curve, *CI* confidence interval, *SEED* Singapore Epidemiology of Eye Diseases study.

Providing the DL algorithm with an increased number of fields of fundus photography similarly showed better performance. Comparing 2-field with 1-field in SiDRP dataset, AUC (0.936 vs 0.908; *P* < 0.001), sensitivity (90.5% vs 89.4%) and specificity (91.9% vs 89.4%) were higher for the former. Examples of the effect of fundus field of views on outcome are represented in Fig. 2d. This trend is similarly seen in the AFEDS dataset as the AUC (0.949 vs 0.911 vs 0.895), sensitivity (90.0% vs 82.6% vs 78.4%) and specificity (86.5% vs 84.4% vs 86.1%) improved when using 7-field images compared to 2-field and 1-field images respectively. Overall, this shows that the DL model’s performance was best for the 7-field, followed by 2-field then by 1-field input images.

Previous cataract surgery showed improvement in the DL algorithm’s ability to detect DR in the pseudophakic eyes compared to phakic eyes as AUC (0.918 vs 0.833; *P* < 0.001), sensitivity (93.4% vs 91.1%), specificity (84.2% vs 76.1%) were remarkably higher. It is of note that the specificity of detecting DR in phakic eyes falls below 80%, representing a large number of false positives, non-pathological images misclassified to be referable DR.

### Heatmaps

**Fig. 3:**
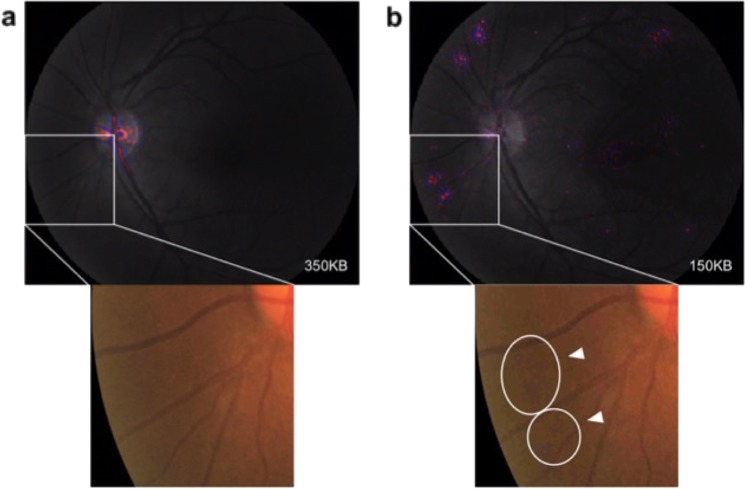
Heatmaps generated for compressed images. Heatmaps showing the ‘hot’ areas that the DL algorithm focuses its attention on when making a diagnostic assessment on the retinal image. This was created using the Integrated Gradient method^66^. The colors on the greyscale retina image show the region of interest, with the red showing peak areas of region of interest while the blue shows the background areas of the region of interest. The white box isolates an area of the image to illustrate the difference between images of 350 and 150 KB in size. **a** A fundus photo of a healthy retina that was provided to the DL model as a 350 KB image. This was correctly classified by the DL model as a healthy retina with no DR. The heatmaps show focus on the normal optic disc and vasculature. **b** The same healthy retina is shown but compressed into a 150 KB size. This was misclassified by the DL algorithm as a retina with referable DR. The heatmaps show other regions of interest aside from the normal optic disc. The magnification of one of these anomalous regions of interest depicts pixelations as identified by the white arrows and ovals. These pixelations amalgamate into a mistaken pathological manifestation of DR, resulting in its false positive status.

Subsidiary heatmap analysis was conducted to explore the rationale for the decrease in performance observed in images of increasing compression. An example of the heatmaps is shown in Fig. 3. This displayed a heatmap of a healthy retina that was accurately classified by the DL model as having no DR when the original 350 KB image was provided. Once provided with the compressed image of 150 KB in size, this was falsely classified as positive for referable DR. The heatmap showed that pixelation of the retina caused by the Joint Photographic Experts Group (JPEG) lossy compression was perceived by the DL algorithm as a pathological manifestation of DR, thus resulting in the conversion from a true negative case to a false positive case.

## Discussion

Our study provides insights that are useful for the development of DL algorithms for detecting DR from retinal photographs. Overall, for DR detection from retinal images, technical factors (CNN and computational framework) do not appear to impact on diagnostic performance of the DL algorithm, but image-related factors (e.g., image compression, number of fields, prior cataract surgery) had a greater and significant impact. First, our study shows that it is indifferent to utilize different CNNs and computational frameworks to build the DL algorithm, as all show comparable diagnostic performance in detecting referable DR (AUC, sensitivity and specificity >0.90). Although combining three CNNs into an Ensemble model yielded a statistically higher performance (AUC 0.944 vs 0.938), this may not be clinically significant. Second, reduction in image size below 250 KB results in significantly lower performance of the DL algorithm, especially reducing specificity to 88.8, 85.3, 72.4%. From the heat map analysis, compressed retinal images with lower image size were more pixelated and had more activity areas, although the changes were not obvious on the color photographs. Third, the performance of DL algorithm showed improved performance with increased number of fields (7-field is more superior than 2-field than 1-field), demonstrating the importance of covering more retina areas for DR screening. Fourth, lens status has important implications, with pseudophakic eyes associated with improved diagnostic performance when compared to phakic eyes.

Our study further supports existing literature demonstrating that utilization of newer CNNs with increasing complexity does not greatly improve the performance of DL algorithms^35^. This is with the exception of an Ensemble of multiple networks which often demonstrated superior results^24,36^. Previous studies examining different computational frameworks in the accuracy at general image classification tasks also showed comparable performance^37,38^. Although there have not been specific studies addressing the effect of compression of retinal images in the context of DL algorithms detection of DR, our study reinforces previous studies that have demonstrated the robustness of DL models with compression of general non-medical images up to a compression threshold^23^.

Possible explanations for our findings are as follows. Advances in DL methods have made it possible to exceed human performance with error rates below 5%^39^. CNNs that belong to this era include ResNet and DenseNet, with VGGNet falling close behind^40,41^. It could be postulated that changes in DL model architecture may not affect the performance significantly because the limiting factor is the quality information the input images provide. To some extent, heatmaps provided the rationale behind the performance observed when utilizing compressed images. Increasing compression resulted in lower resolution to an extent where the image may not hold enough information to distinguish hemorrhages from the background, thus causing a decrease in sensitivity from 90.5 to 83.5%. It may also result in increasing distortion of the picture where normal retina or vascular architecture may be misinterpreted as pathological manifestations of DR such as hemorrhages or venous bleeding, causing a decrease in specificity^42^. United Kingdom national screening guidelines recommends retinal images to be compressed to no less than 400KB, implying the importance of image size in adequate assessment of DR^43^.

It is apparent that a greater view of the retina allows for more accurate diagnosis due to an increase in information^44,45^. Therefore, this would explain the findings that when provided with only 1-field, the DL models’ performance dropped (from AUC 0.911 to 0.895) and why when provided with 7-fields, the performance improved (from AUC 0.911 to 0.949). With additional evidence that suggest an estimated 30% of lesions located around the Early Treatment Diabetic Retinopathy Study (ETDRS) 7-fields, this further supports the improvement in increased number of fields^46^. However, it is interesting to note the high performance of the DL algorithm despite providing only 1-field. Our study shows that the DL system yielded best performance on 7-field retinal images, although this may not be practical to do that in the routine setting as it requires pupil dilation, experienced photographers and patients’ compliance. Despite this, this DL system showed clinically acceptable outcome (AUC > 0.90) on 1-field and 2-field photographs for DR screening. A possible reason for this high performance could be due to the distribution of manifestations of DR important for diagnosis. This is because some studies report a skewed topological distribution of DR lesions concentrating in the areas lateral to the macula and in the temporal retina, those areas visible to the macula-centered image^47,48^. It should be noted that there is a possible confounding factor of increased number of fields providing increased number of images per eye, thus providing a better result. Phakic lens status and cataract, with resultant impact on media opacity and the gradability of retinal images would also result in a decrease in specificity from the increased false positives observed and this supports the better performance reported in pseudophakic eyes^49^.

There is an increasing desire for research groups around the world to develop their own DL algorithms tailored to their specific purpose with aspiration to emulate the successes of previously published DL algorithms. In addition, many clinicians, healthcare professionals and policymakers making decisions on the adoption of a DL algorithms increasingly require a comprehensive guide on the clinical translatability of these algorithms in the specific clinical context they operate in. This paper provides a broad guidance in the technical and image-related factors that should be considered during the development and deployment of DL models, concentrating on factors that would vary based on the intended purpose of the DL models and the resources available. Subsequent investigations may consider expanding on this study to explore the effect of altering other factors governing the characteristics of the input images such as comparisons between a myriad of fundus cameras: non-mydriatic and mydriatic, table top and handheld, color and mono-chromatic. This is due to the finding that specifications of input images may be deemed to be the main limiting factor to improving the algorithm’s performance.

Our current study has several limitations which should be acknowledged. The analysis of the following five described factors: choice of CNN, computational framework, image compression, field of view, and previous cataract surgery is not exhaustive. There are other computational frameworks (e.g., PyTorch, CNTK), CNNs (e.g., Inception, AlexNet), and variations in number of fundus fields of view (e.g., wide and ultrawide field retinal imaging) that were not included in this analysis. Inclusion of these would not be pragmatic given the wide range of technologies currently available with novel ones being introduced frequently. In our investigation into the features of the input image and the effect on performance, we considered factors that would be clinically relevant and beneficial for real-world applications^50^. For example, the potential of DL to expand coverage of healthcare services to rural areas with limited access required compromise on the image size and the number of fields captured per eye for screening purposes to compensate for limited economic resources such as manpower, data storage, and connectivity. Although our study shows that the DL performed better in the pseudophakic than phakic eyes, this may not change how we would screen for patients with diabetes. In addition, the patients with early cataract could still have clear media to allow good quality retinal images, although we do not have such information captured in our study.

Another limitation is this study focused only on DR detection from fundus imaging, thus the findings may not be applicable to other ocular conditions and imaging modalities. Nevertheless, we feel that the technical and image-related factors that we have identified as important in this study may be extrapolated to DL algorithms being applied for diagnosis of other ocular diseases from retinal images. The relative importance of these factors when applied to detection of other ocular diseases, or using alternative imaging modalities (such as optical coherence tomography) is an interesting area for further study.

In conclusion, our study provides a guide for researchers to understand the factors that may impact the development of DL algorithms for detection of DR and other conditions from retinal photographs, particularly when using images from real-world populations. Various image-related factors play more significant roles than technical factors in determining the diagnostic performance, suggesting the importance of having robust training and testing datasets for DL training and deployment in the real-world settings. In order to ensure a successful translation of a DR screening algorithm, it is important to consider technical factors (e.g., types of CNN, computational framework) and image-related factors (e.g., compression levels, number of fields, media clarity, mydriatic status, retinal cameras, pigmentation of different races, disease prevalence, systemic vascular risk factors, concurrent ocular diseases and reference standards).

## Methods

We first conducted a systematic literature review on factors affecting DL algorithms in detection of DR using search engines PubMed, Web of Science, Scopus, ProQuest and IEEE Xplore searching for peer-reviewed studies up to 20 September 2019. Keywords used were ‘diabetic retinopathy’, ‘deep learning’, ‘technical factor’ and ‘image-related factor’.

Based on the systematic literature review (Fig. 1), we then selected the following factors to conduct the following analyses. First, we looked at different CNNs within the DL algorithm architecture: VGGNet, ResNet, DenseNet and Ensemble (a combination of the aforementioned three CNNs). Second, we evaluated the impact of altering the computational framework used to implement the coded CNNs: Caffe and TensorFlow. Third, we analyzed the effect of different image sizes generated through the process of compression, specifically looking at the five image sizes 350 (original), 300, 250, 200, 150 KB. Fourth, we looked at various numbers of input field of views captured per eye: 1-field macula-centered image, 2-field macula-centered and optic disc-centered images, 7-field ETDRS standard fields. Fifth, we looked at the impact of eyes with prior cataract surgery compared to eyes with cataract on the ability of the DL algorithm to accurately detect DR.

### Study population dataset: training dataset

The DL algorithm was trained to detect referable DR with 76,370 retinal fundus photographs obtained from 13,099 patients from the database of SiDRP between 2010 and 2013. The definition of DR was made based on the International Classification Diabetic Retinopathy Severity Scale (ICDRSS) (Supplementary Fig. 1). SiDRP is a national screening program in Singapore established in 2010 which utilizes a tele-ophthalmology platform where fundus photographs captured in primary care clinics are transmitted to a centralized team of trained graders^15,51^. The 45 degree angle retinal fundus photographs are all taken with Topcon TRC-NW8 Non-Mydriatic Retinal Cameras in two fields of view per eye, an optic disc-centered image and a macula-centered image, with both eyes taken per patient. These two-field images in the training dataset were notably 350 KB average in size each. This training dataset included phakic and pseudophakic eyes. One round of training was conducted with no further re-training of the algorithm. For the purposes of collating a robust training dataset for the DL algorithms, two senior certified non-medical graders with more than five years’ experience were tasked to grade each eye. Discordant grades between the two graders were arbitrated by a retinal specialist. Poor quality images deemed ungradable to senior non-medical professional graders were removed from the training and testing dataset, especially if the images had more than a third of the photograph obscured. These images were not used in the analysis of the DL algorithm.

### Technical factors: different CNNs

**Fig. 4:**
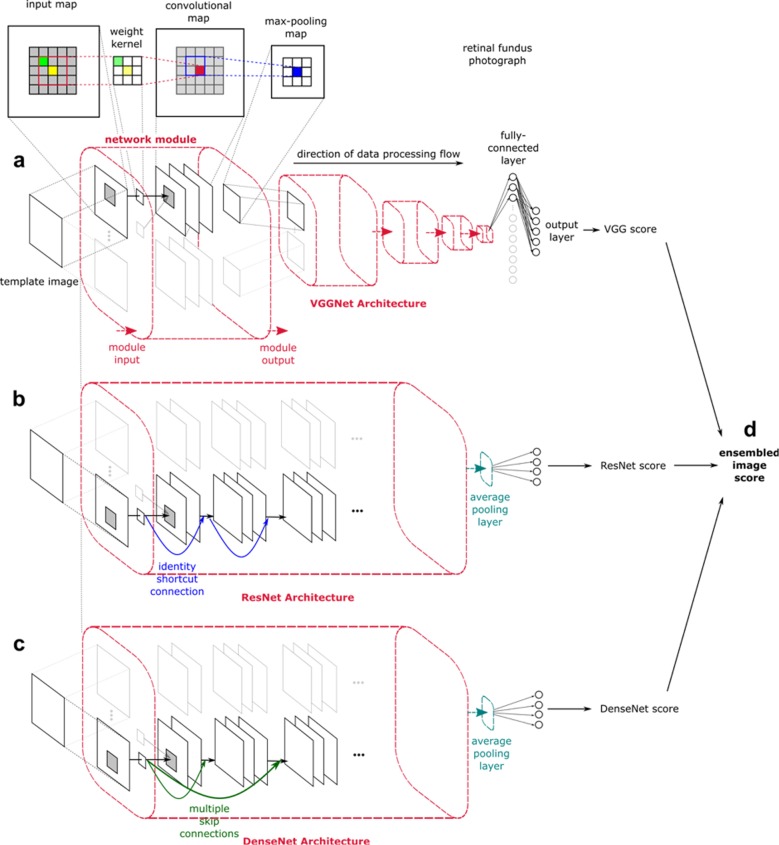
Convolutional neural networks investigated. The architecture of convolutional neural networks (CNNs) are based on few general principles. The network is composed of mathematically weighted neurons that form sequential layers where there is linear transfer of signal from the input through to the output layers. For this study, each input image was pre-processed by scaling to a fixed template of 512 × 512 pixels in resolution. These images were subsequently represented as a matrix of Red Green Blue (RGB) values in the input layer. Sequential convolutions were conducted by superimposing a weighted kernel over these input maps, with our study using a 3 × 3 weighted kernel with subsequent max-pooling. The output layer utilizes a softmax classifier to generate probability values for the pre-defined output classes^15,32,52^. **a** VGGNet is the oldest CNN used in this comparison, released in 2014. Despite its standard uniform architecture composed of 16 layers, it has had great success at feature extraction^53^. **b** ResNet has been highly favored since its introduction in 2015, with its atypical architecture utilizing skip residual connections (visualized as blue arrows) to bypass signals across layers. This allows for increase in layers without compromising the ease of training, resulting in supra-human performance of 3.6% top-5 error rate^54^. **c** DenseNet is a newer CNN released in 2017 that has been shown to perform better than ResNet. Its architecture builds on a similar principle to the one capitalized by ResNet, but rather has a dense connectivity pattern where each layer receives information from all preceding layers as shown by the green arrows. This allows concatenation of sequential layers and compacting the network into a ‘denser’ configuration^40^. **d** Ensemble is a combination of the three networks’ probability output scores generated per eye, through the acquisition of the mean value.

Three CNNs were explored in this study, namely VGGNet, ResNet and DenseNet. Additionally, the three CNNs were further combined into an Ensemble model. The CNNs were built using TensorFlow with Keras API specification (Fig. 4). Pre-trained models with transfer learning were available in the Keras library. All models had a final max-pooling layer added before the output layer.

1. VGGNet: This is a 16-layered network, designed by the Visual Geometry Group in Oxford in 2014. VGGNet has been popular due to its excellent performance on the classification of retinal images^15,52,53^. An overview of the architecture can be seen in Fig. 4a.
2. ResNet: For this study, we used ResNet-50 consisting of 50 layers which surpassed human performance with an error rate of 3.6%^54^. Recently, it has been widely used to detect age-related macular degeneration, diabetic macular edema, and glaucoma^24,55,56^. ResNet’s rise in popularity is attributed to its ability to increase depth of the network architecture through ‘skip’ residual connections equipped to perform identity mappings, thus increasing accuracy whilst still being easy to train (Fig. 4b).
3. DenseNet: This CNN consisted of 121 layers densely connected through concatenating sequential layers in a feedforward fashion to achieve increased depth of deep CNNs efficiently (Fig. 4c)^40^.
4. Ensemble: Ensemble consists of the above three networks’ (VGGNet, ResNet, and DenseNet) and its output is established as an average over outputs of the component networks per eye. Performance is expected to match or exceed single CNNs (Fig. 4d)^57^.

### Technical factors: different computational frameworks

The DL algorithms were then constructed using two open-sourced computational frameworks–Caffe and TensorFlow to compute VGGNet using Python (a programing language).

1. Caffe: This was reconstructed from the reference paper, initialized with Xavier initialization and included an extra module of convolutional and pooling layer to cater for increased resolution of input images to 512 × 512^58^. This VGGNet Caffe DL algorithm was the control architecture for analysis of the image-related factors.
2. TensorFlow: In this study, TensorFlow was implemented with Keras API specification. In addition, a single pooling layer preceding the output layer in VGGNet was added^59^.

### Image-related factors: different image compression

Increasing compression may allow ease of transmission in tele-ophthalmology but may compromise image quality. To investigate the effect of input image compression on performance, 71,896 original images (35,948 eyes of 14,880 patients) taken from SiDRP between 2014 and 2015 were used as the archetype to create five distinct sets of fundus images with different compression levels. These were 45 degree angle fundus fields taken with Topcon TRC-NW8 Non-Mydriatic Retinal Cameras. In total, 359,480 retinal fundus images were generated. These five sets were then used to test the DL algorithm. It should be noted that the DL algorithm was previously trained on the original 350KB images. These images were compressed from an average of 350 KB JPEG images to four additional levels of compression, averaging 300, 250, 200, 150 KB in image size respectively. This was achieved with the use of a standard JPEG compression algorithm using the Independent JPEG Group’s library of quality levels. We used a publicly available algorithm on the OpenCV library.

### Image-related factors: different fundus field of view

We evaluated the effect of different fundus field of views on the performance of the DL algorithm by comparing (1) 1-field macula-centered and (2) 2-field optic disc and macula-centered fundus photographs. Both subsets were taken from data collected from SiDRP between 2014 to 2015 containing 35,948 eyes. Macula-centered or optic disc-centered is defined as the macula or the optic disc, respectively, located less than one disc diameter circumferentially from center of the image. In addition, we also looked at increasing number of fields, comparing (1) 1-field, (2) 2-field, and (3) 7-field fundus imaging. For this analysis, we used an external testing dataset from the AFEDS that obtained the ETDRS stereoscopic reference standard of 7-field fundus imaging^60,61^. 7-field, 2-field, and 1-field retinal images collected from a fixed set of 1403 eyes in this dataset were used. A total of 9821 images were used.

### Image-related factors: previous cataract surgery

To assess the impact of previous cataract surgery on the ability for the DL algorithm to detect DR on fundus photographs, we employed an external testing dataset using retinal images from participants of SEED Study (baseline, 2004-11). The SEED study is a population-based epidemiologic study that comprised three major ethnic groups in Singapore – Malay patients were recruited from 2004 to 2006, Indian patients from 2007 to 2009, and Chinese patients from 2009 to 2011^62–65^. Phakic and pseudophakic eyes in this dataset were separated and analysis was conducted to compare one group with relation to the other. Among the 4910 eyes (9820 images) included, 1612 eyes were phakic and 3298 eyes were pseudophakic.

### Heatmap

Heatmaps were generated to provide insight into the conundrum of DL, the black box of learning, as they demonstrate focus areas visualized by the DL system. The method of Integrated Gradient was used to generate these heatmaps^66^.

### Reference standard of testing dataset

The reference standard of the severity of DR of each eye in the SiDRP and SEED testing datasets was set as the grading assessment by an ophthalmologist sub-specializing in retinal diseases, with over five years’ experience in assessing DR. For the AFEDS testing dataset, concurring assessments from two retinal specialists were used as the reference standard. The grading was conducted in accordance to ICDRSS. However, for the purposes of this study, we reclassified this scale to a binary outcome measure of referable DR or non-referable DR. Referable DR is defined as moderate non-proliferative DR or worse, including diabetic macular edema.

### Statistical analysis

We used the following primary outcome measures as a marker for DL algorithm’s performance in detecting referable DR: AUC, sensitivity and specificity of the algorithm at detecting DR with respect to the reference standard. The operating thresholds were pre-set during training of each modification of the DL algorithms’ technical parameters. During training, AUCs were determined for the training dataset while sensitivities and specificities were calculated across a range of thresholds. The optimal threshold for each DL algorithm was chosen to achieve 90% sensitivity. The 95% confidence intervals (CI) for sensitivity and specificity were calculated with the exact Clopper-Pearson method and for AUC, empirical bootstrap with 5000 replicates was used. To evaluate statistical significance of difference in results for comparison of AUCs, the empirical bootstrap with 5000 replicates was used. All statistical analyses were performed using the R statistical software (version 3.5.1; R Foundation for Statistical Computing, Vienna, Austria). P value less than 0.05 was considered statistically significant.

### Ethics approval

Our study was approved by the centralized institutional review board (IRB) of SingHealth, Singapore (IRB reference number 2018/2433). It was conducted in accordance with the Declaration of Helsinki. Informed consent by the patients were exempted by the IRB because it used fully anonymized images retrospectively.

### Reporting summary

Further information on research design is available in the Nature Research Reporting Summary linked to this article.

## Supplementary information

Reporting Summary

Supplementary Information

## Data Availability

The datasets used in this study originated from different principal investigators from different countries. Upon request, the corresponding author D.S.W.T. can send the data request to the individual principal investigator to seek clearance from them.

## Code availability

The artificial intelligence system described in this study is kept at the Singapore Eye Research Institute (SERI) and National University of Singapore (NUS). The underlying algorithm is copyrighted by SERI, NUS and will not be available to public.
