# Supplementary material for: Technical and imaging factors influencing performance of deep learning systems for diabetic retinopathy
Source: NPJ Digit Med. 2020 Mar 23;3:40. doi: 10.1038/s41746-020-0247-1 (PMC7090044; doi:10.1038/s41746-020-0247-1)
Supplement: Supplementary file 2 — Supplementary Information [file 41746_2020_247_MOESM2_ESM.pdf]

SUPPLEMENTARY INFORMATION

Supplementary Figure 1. Retinal Fundus Photographs Depicting Stages of DR

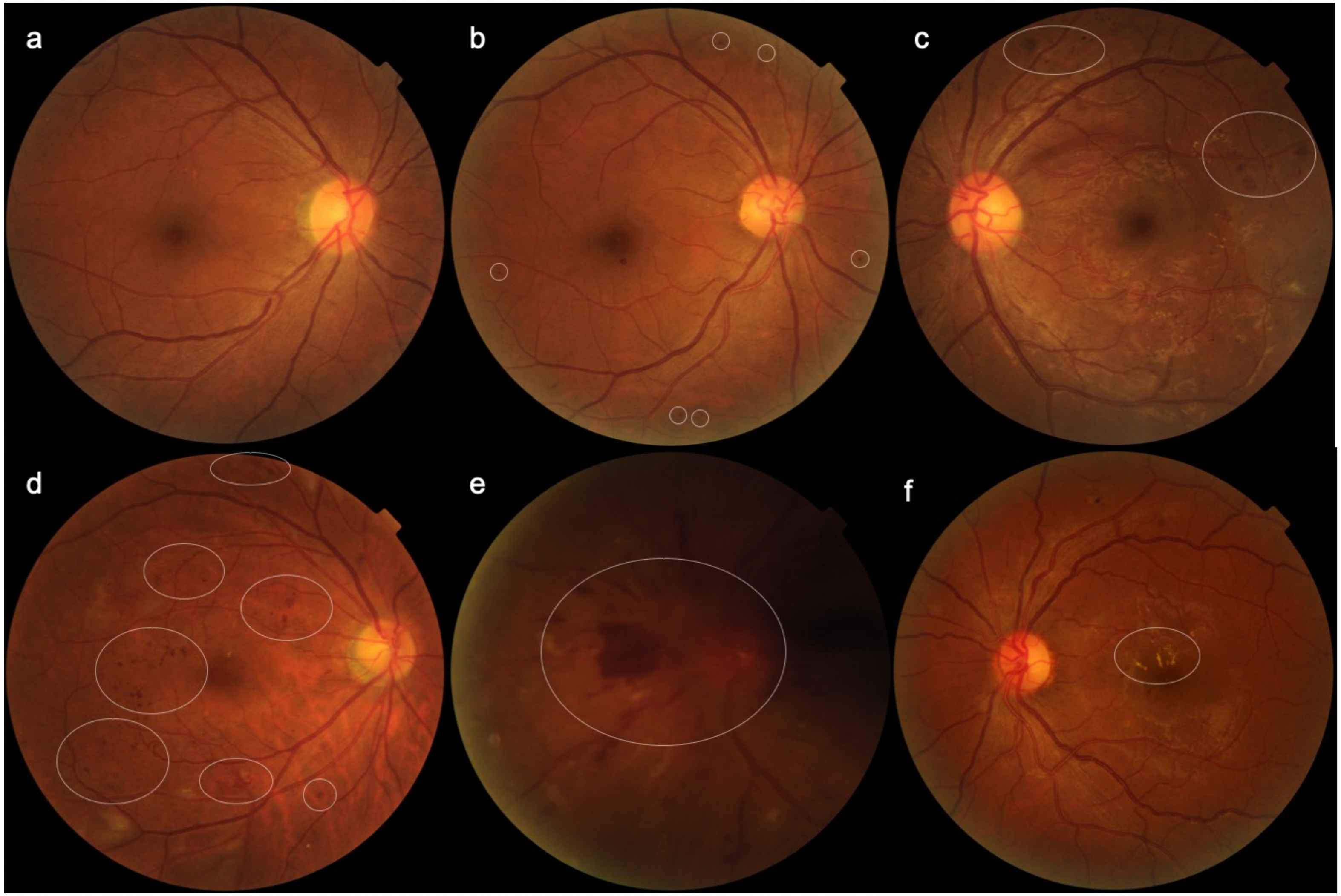

Retinal fundus images depicting the stages of Diabetic Retinopathy (DR) severity as defined by the International Classification of Diabetic Retinopathy Severity (ICDRS) Scale. The white ovals are image markers for identification of lesions that support the diagnosis of the fundus images' DR severity. **a.** No DR. **b.** Mild Non-Proliferative DR (NPDR). White ovals annotate microaneurysms. **c.** Moderate NPDR. White ovals denote intra-retinal haemorrhages. **d.** Severe NPDR. White ovals demonstrate more than 20 intra-retinal haemorrhages are present in all 4 quadrants of the retina. **e.** Proliferative DR. The annotated white oval show a large pre-retinal haemorrhages nasal to disc from new vessels elsewhere (NVE), with some new vessels on the disc (NVD) **f.** Diabetic Macular Oedema. The white oval demarcates the presence of exudates within 1 disc diameter from the fovea.
